# Progression of Primary Cutaneous Cryptococcosis to Disseminated Cryptococcosis in an Immunosuppressed Older Patient: A Case Report

**DOI:** 10.7759/cureus.105903

**Published:** 2026-03-26

**Authors:** Mina Maruta, Moe Kyotani, Tsuneaki Kenzaka

**Affiliations:** 1 Department of Internal Medicine, Hyogo Prefectural Tamba Medical Center, Tamba, JPN; 2 Division of Community Medicine and Career Development, Kobe University Graduate School of Medicine, Kobe, JPN

**Keywords:** case report, corticosteroids, cryptococcus neoformans, cutaneous infection, disseminated cryptococcosis, immunosuppression, primary cutaneous cryptococcosis

## Abstract

Primary cutaneous cryptococcosis (PCC) is acquired through direct inoculation of *Cryptococcus neoformans* into wounds or damaged skin, typically resulting in a localized infection confined to a single cutaneous site. Progression of PCC to disseminated cryptococcosis is extremely rare. The purpose of this case report is to describe an unusual instance of PCC progressing into disseminated disease in an immunosuppressed older patient and to highlight the importance of considering cryptococcosis as a differential diagnosis of cutaneous lesions unresponsive to antimicrobial therapy.

We report the case of an 88-year-old woman with Evans syndrome receiving oral prednisolone (17.5 mg/day) who also had diabetes mellitus and engaged in home gardening. She developed redness, swelling, and induration of the right forearm 18 days before admission and was treated with cefazolin for presumed cellulitis. Subsequently, yeast-like fungi were detected from the wound and blood cultures on hospital day 7, and *C. neoformans* was identified on hospital day 8, leading to the diagnosis of disseminated cryptococcosis. Antifungal therapy with amphotericin B (150 mg/day) and flucytosine (2.5 mg/day) was initiated the same day. *C. neoformans* was later isolated from cerebrospinal fluid. Despite antifungal treatment, her condition progressively deteriorated, and she died.

This case illustrates an exceptionally rare progression of PCC to disseminated disease in an older immunosuppressed patient. It highlights the need for heightened clinical suspicion of cryptococcosis when cutaneous lesions fail to respond to antimicrobial therapy, particularly in individuals receiving corticosteroids or with additional risk factors such as diabetes or soil exposure. Early recognition and appropriate antifungal management are essential to prevent fatal outcomes.

## Introduction

*Cryptococcus neoformans* (*C. neoformans*) is a yeast-like fungus commonly found in environmental sources, particularly soil [1]. It thrives in bird droppings, especially those of pigeons, and can infect humans through inhalation of airborne spores or via direct inoculation through skin wounds. *C. neoformans* is a major cause of opportunistic infections in immunocompromised individuals. Most cutaneous lesions occur secondary to involvement following pulmonary infection, with hematogenous spread leading to multiple skin lesions [2].

Primary cutaneous cryptococcosis (PCC) is rare and arises from direct inoculation through damaged skin, typically presenting as a single cutaneous site [3]. Known risk factors for PCC include steroid use and diabetes mellitus [4]. Diabetes mellitus impairs host immunity through multiple mechanisms, including reduced neutrophil chemotaxis and phagocytic activity, impaired intracellular killing, and defects in cell-mediated immunity, all of which increase susceptibility to fungal infections such as cryptococcosis [5]. In most reported cases, PCC remains localized, and progression to disseminated cryptococcosis is considered extremely uncommon. In fact, progression from PCC to disseminated cryptococcosis is exceedingly rare; Christianson et al. reported only 2 disseminated cases among 28 PCC cases [4], and Patil et al. identified just 1 disseminated case in a review of 73 PCC cases [6]. Moreover, most published studies provide limited detail regarding the clinical circumstances under which such progression occurs.

Existing literature has primarily focused on differentiating PCC from secondary cutaneous involvement in disseminated cryptococcosis and on describing its clinical manifestations and treatment outcomes. However, a notable gap remains regarding the specific host factors and environmental exposures that predispose PCC to systemic dissemination, particularly in older immunosuppressed patients without HIV infection or organ transplantation. The combined influence of moderate-dose corticosteroid therapy, diabetes mellitus, and soil exposure has not been well described as a constellation of risk factors for PCC progression, although each of these factors is individually recognized as a predisposing condition for cryptococcal infection [4-7]. Specifically, existing studies rarely provide a detailed characterization of the host immune status, environmental exposures, and temporal clinical evolution preceding dissemination, representing a gap that the present case helps to address.

Herein, we report an exceptionally rare case of PCC that progressed to disseminated cryptococcosis in an immunosuppressed older patient receiving oral prednisolone. The purpose of this case report is to address the existing gap by detailing the clinical course of this unusual progression and to emphasize the importance of considering cryptococcosis as a differential diagnosis of cutaneous lesions unresponsive to antimicrobial therapy in immunosuppressed individuals.

## Case presentation

An 88-year-old Japanese woman with a medical history of Evans syndrome treated with oral prednisolone (17.5 mg/day), type 2 diabetes mellitus, angina pectoris, abdominal aortic aneurysm, and hypertension presented with a progressively worsening skin lesion on her right wrist. She engaged in home gardening but reported no contact with birds. Eighteen days before admission, she noticed a 3 × 3-cm indurated and erythematous lesion on the dorsal aspect of her right wrist, which gradually extended to her right forearm. She consulted a dermatologist 10 days prior to hospitalization and was treated with gentamicin ointment and gauze protection; however, the rash progressed to the area around her right elbow, resulting in impaired movement. At that time, this extension was considered a contiguous local spread from the initial wrist lesion rather than new primary lesions or hematogenous dissemination, as no systemic symptoms or distant cutaneous lesions were present. Consequently, she was referred to our hospital. At presentation, she was diagnosed with cellulitis, and inpatient treatment was initiated. This initial diagnosis was supported by several clinical features: the lesion showed progressive erythema, swelling, warmth, and tenderness-findings typical of bacterial cellulitis-and there was no evidence of systemic symptoms, distant skin lesions, or neurological abnormalities at that time. Additionally, the burn‑like appearance and localized extension pattern were more suggestive of a bacterial soft‑tissue infection than a fungal etiology. At the time of presentation, early disseminated cryptococcosis was considered unlikely because the patient exhibited no systemic symptoms such as fever, headache, or altered mental status, and no distant or non-contiguous cutaneous lesions were present. The skin findings showed a contiguous extension from the initial wrist lesion rather than multiple lesions suggestive of hematogenous spread. Additionally, laboratory results did not indicate systemic inflammation. These features supported the initial impression of a localized process rather than early disseminated disease.

**Figure 1:**
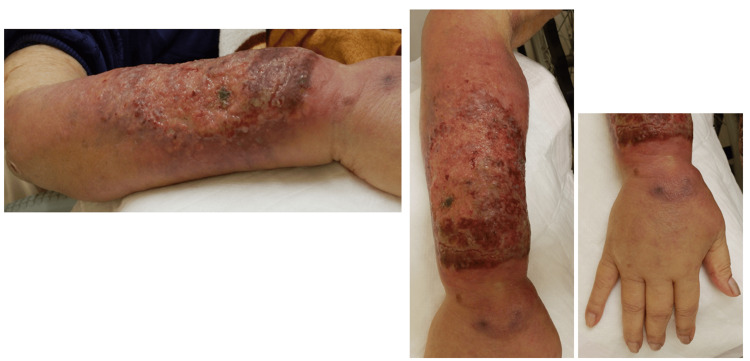
Photograph of the right forearm. A palm-sized erosive and ulcerative lesion is present on the right forearm. Erythema and swelling extended proximally toward the elbow joint. Marked edema extended to the fingers.

On presentation, vital signs were as follows: Glasgow Coma Scale score [8], 15 (eye opening, 4; verbal response, 5; motor response, 6); body temperature, 36.1°C; peripheral oxygen saturation, 99% on room air; blood pressure, 117/75 mmHg; and respiratory rate, 16 breaths per minute. Physical examination revealed no nuchal rigidity, pallor of the palpebral conjunctiva, icterus of the bulbar conjunctiva, and palpable cervical lymphadenopathy. Chest examination revealed clear breath sounds and regular heart sounds without murmurs. The abdomen was flat and soft, non-tender, and without hepatomegaly or splenomegaly. Additionally, a rash with swelling, erythema, erosion, and ulceration was observed on the right forearm (Figure 1).

The results of laboratory testing are shown in Table 1 and included a white blood cell count of 5590/μL with 98.4% neutrophils, C-reactive protein of 3.93 mg/dL, a blood glucose of 135 mg/dL, and hemoglobin A1c of 6.5%.

**Table 1:** Laboratory data upon admission.

Parameter	Recorded value	Standard value
White blood cell count	5590/µL	4500–7500/µL
Neutrophils	98.4%	42–74%
Lymphocytes	1.1%	18–50%
Monocytes	0.5%	1–10%
Hemoglobin	10.9 g/dL	11.3–15.2 g/dL
Mean corpuscular volume	100 fL	82–101 fL
Platelet count	19 × 10^4^/µL	13–35 × 10^4^/µL
D-dimer	4.5μg/mL	<1.0μg/mL
C-reactive protein	3.93 mg/L	≤0.60 mg/dL
Total protein	5.8 g/dL	6.9–8.4 g/dL
Albumin	3.2 g/dL	3.9–5.1 g/dL
Total bilirubin	3.0 mg/dL	0.2–1.2 mg/dL
Aspartate aminotransferase	33 U/L	11–30 U/L
Alanine aminotransferase	32 U/L	4–30 U/L
Lactase dehydrogenase	484 U/L	109–216 U/L
Creatine kinase	28 U/L	40–150 U/L
Blood urea nitrogen	47.3 mg/dL	8–20 mg/dL
Creatinine	1.22 mg/dL	0.63–1.03 mg/dL
Sodium	141 mEq/L	136–148 mEq/L
Potassium	5.0 mEq/L	3.6–5.0 mEq/L
Chloride	103 mEq/L	98–108 mEq/L
Calcium	9.3 mg/dL	8.8–10.1 mg/dL
Glucose	135 mg/dL	70–109 mg/dL
Hemoglobin A1c	6.5%	5.6–5.9%

**Figure 2:**
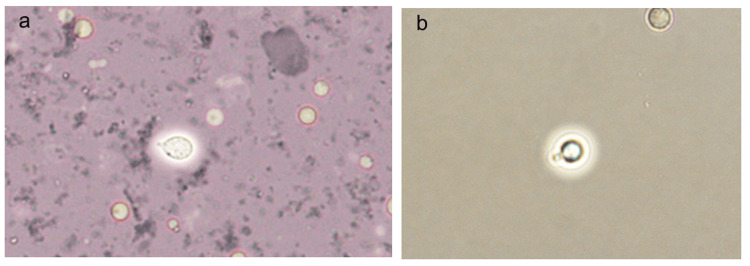
(a) India ink staining (blood culture), ×2000. (b) India ink staining (cerebrospinal fluid culture), ×2000. Yeast-like fungi surrounded by a thick capsule were detected in both blood cultures and cerebrospinal fluid cultures.

The skin lesion exhibited an appearance suggestive of a burn-like dermatosis. Suspecting a staphylococcal infection, we initiated cefazolin at a dose of 2 g/day; however, the lesion showed minimal improvement. Subsequently, yeast-like fungi were detected from the wound and blood cultures on hospital day 7, raising suspicion for *Cryptococcus species*. Because species identification was not yet available at that time, antifungal therapy was deferred. On hospital day 8, *C. neoformans* was identified, leading to the diagnosis of disseminated cryptococcosis, and antifungal therapy with amphotericin B (150 mg/day) and flucytosine (2.5 mg/day) was initiated immediately (Figure 2). On hospital day 9, the patient developed impaired consciousness. Immediately after the onset of impaired consciousness, non-contrast head computed tomography was performed, which revealed no acute abnormalities. Given the absence of radiological findings, cerebrospinal fluid analysis was subsequently conducted, and cryptococcal antigen was detected.

*C. neoformans* were isolated from cultures of the skin, blood, and cerebrospinal fluid (Table 2). The identified species and antifungal susceptibility profiles were identical across all three sites. Despite antifungal therapy, the rash spread to other areas, including the lower extremities, and her level of consciousness showed minimal improvement.　

**Table 2:** Antifungal susceptibility results of Cryptococcus neoformans isolated from skin, blood, and cerebrospinal fluid cultures MIC (minimum inhibitory concentration) values represent the lowest concentration of each antifungal agent that inhibits visible growth of Cryptococcus neoformans. Lower MIC values indicate higher susceptibility. The MIC values obtained from skin, blood, and cerebrospinal fluid cultures were identical in this case.

Antifungal agent	Skin culture MIC (µg/mL)	Blood culture MIC (µg/mL)	Cerebrospinal fluid culture MIC (µg/mL)
Flucytosine	<=4	<=4	<=4
Amphotericin B	<=0.125	<=0.125	<=0.125
Fluconazole	<=2	<=2	<=2
Itraconazole	<=0.015	<=0.015	<=0.015
Miconazole	<=0.03	<=0.03	<=0.03
Micafungin	<=16	<=16	<=16
Voriconazole	<=0.015	<=0.015	<=0.015

**Figure 3:**
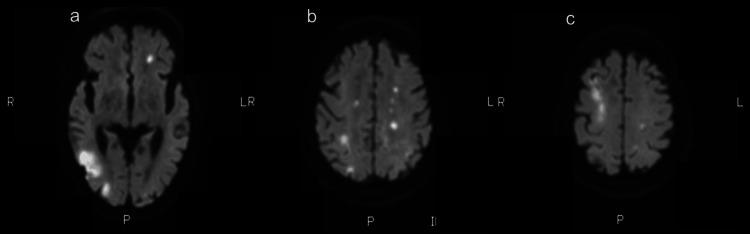
Head magnetic resonance imaging (diffusion weighted imaging). (a) Diffusion-weighted axial MRI at the basal ganglia level shows multiple hyperintense lesions consistent with acute ischemic infarctions. (b) Diffusion-weighted axial MRI at the centrum semiovale level demonstrates scattered punctate hyperintense lesions in both cerebral hemispheres. (c) Diffusion-weighted axial MRI at the cortical level reveals additional multifocal diffusion-restricted lesions involving the cerebral cortex. These findings are suggestive of multiple cerebral infarctions, likely related to an infectious or embolic process in the setting of disseminated cryptococcosis.

By hospital day 16, the patient developed hypotension requiring continuous administration of norepinephrine and dobutamine. Brain magnetic resonance imaging performed the same day revealed multiple cerebral infarctions suggestive of infectious etiology (Figure 3).

Her consciousness and respiratory status continued to deteriorate, and she died on hospital day 27.

## Discussion

We encountered a case of PCC that progressed to disseminated cryptococcosis. PCC arises from direct inoculation of *Cryptococcus* species through disrupted skin and typically remains confined to a single cutaneous site; progression to disseminated disease is exceedingly rare. In this case, oral prednisolone therapy for Evans syndrome was considered a contributing risk factor.

Most cutaneous lesions caused by *Cryptococcus* represent secondary involvement due to disseminated disease, with inhalation of aerosolized microscopic yeast forms being the primary route of infection. Hematogenous dissemination results in the simultaneous appearance of lesions at multiple skin sites [2]. Furthermore, *C. neoformans* is capable of invading the central nervous system, leading to life-threatening conditions such as meningitis or meningoencephalitis, presenting with symptoms including headache, elevated intracranial pressure, fever, and coma [9]. Other sites of hematogenous spread include the bones, joints, kidneys, adrenal glands, spleen, and prostate [2]. Cutaneous manifestations occur in 10-20% of disseminated cryptococcal infections [10].

In contrast, PCC results from direct inoculation through damaged skin and typically involves only a single cutaneous site. It is a rare condition that can occur in both immunocompetent and immunocompromised individuals [6]. The cutaneous manifestations are diverse, including ulceration, vesicle formation, and nodular lesions, often necessitating differentiation from bacterial skin infections such as cellulitis and impetigo [4].

Occupations and hobbies associated with skin trauma or soil exposure are recognized risk factors for PCC [7]. Additionally, factors that impair cellular immunity (such as corticosteroid use, solid organ transplantation, sarcoidosis, diabetes, and other forms of immunosuppression) are known risk factors [4]. In a review of 73 PCC cases, corticosteroid use was the most common risk factor among 37 immunocompromised patients, with a mean daily dose of 15 mg/day (median 13 mg/day) [6]. In the present case, oral prednisolone at a dose of 17.5 mg/day, coexisting diabetes, and the patient’s hobby of home gardening were considered contributing factors and possibly played a role in the development of PCC. In addition to corticosteroid therapy, the patient’s underlying Evans syndrome may also have contributed to her impaired immune function. Evans syndrome is associated with immune dysregulation, and affected individuals often require prolonged immunosuppressive treatment, which further compromises host defense mechanisms [11]. This combination of intrinsic immune dysfunction and steroid-induced immunosuppression may have increased her susceptibility to cryptococcal infection and contributed to the rapid progression to disseminated disease.

Although PCC itself is rare, progression from PCC to disseminated cryptococcosis has been documented only in a small number of cases [4,6]. However, the clinical circumstances under which such progression occurs remain poorly characterized, particularly in older immunosuppressed patients without HIV infection or organ transplantation. Existing reports often lack detailed descriptions of host factors, environmental exposures, and the temporal evolution of cutaneous findings preceding dissemination. This gap limits clinicians’ ability to identify patients at risk for systemic progression.

This case provides valuable insight into this gap by illustrating how moderate-dose corticosteroid therapy (17.5 mg/day), diabetes mellitus, and soil exposure through gardening may collectively predispose an older immunosuppressed patient to PCC and subsequent dissemination. The detailed clinical course (including the initial misdiagnosis as bacterial cellulitis, rapid progression of skin lesions, and subsequent central nervous system involvement) offers a rare and informative example of how PCC can evolve despite early antimicrobial therapy.

This case highlights several important clinical implications: (i) Moderate-dose corticosteroids may still substantially impair cellular immunity, increasing the risk of PCC and its progression to disseminated disease; (ii) Cutaneous lesions unresponsive to antibacterial therapy warrant early evaluation for fungal pathogens, particularly in immunosuppressed individuals; (iii) Environmental exposure such as gardening remains an important risk factor, even in the absence of bird contact; and (iv) Early fungal cultures and cryptococcal antigen testing may prevent diagnostic delay, which is critical given the high mortality associated with disseminated cryptococcosis.

Because this report describes a single patient, the potential combined effect of these risk factors should be interpreted cautiously. Further accumulation of similar cases is needed to clarify their interaction.

## Conclusions

In summary, this case represents an extremely rare instance of PCC progressing to disseminated cryptococcosis and fills an important gap in the literature by detailing the clinical circumstances under which such progression may occur. It underscores the need for heightened clinical suspicion of cryptococcosis in immunosuppressed patients presenting with atypical or treatment-refractory cutaneous lesions and emphasizes the importance of early diagnostic evaluation and appropriate antifungal management.
